# Evaluation of the nicotine metabolite ratio in smoking patients treated with varenicline and bupropion

**DOI:** 10.3389/fphar.2022.900112

**Published:** 2022-07-22

**Authors:** Paulo Roberto Xavier Tomaz, Thuane Sales Gonçalves, Juliana Rocha Santos, Jaqueline Scholz, Tânia Ogawa Abe, Patrícia Viviane Gaya, Eduardo Costa Figueiredo, Henrique Dipe de Faria, Isarita Martins, Ana Miguel Fonseca Pego, Beatriz Aparecida Bismara, Maurício Yonamine, Alexandre Costa Pereira, Paulo Caleb Júnior Lima Santos

**Affiliations:** ^1^ Laboratory of Genetics and Molecular Cardiology, Instituto do Coracão (InCor), Hospital das Clinicas HCFMUSP, Faculdade de Medicina, Universidade de São Paulo, São Paulo, Brazi; ^2^ Laboratory of Clinical Pharmacology and Translational Research, Department of Pharmacology, Escola Paulista de Medicina, Universidade Federal de São Paulo EPM-Unifesp, São Paulo, Brazil; ^3^ Smoking Cessation Program Department, Instituto do Coracão (InCor), Hospital das Clínicas HCFMUSP, Faculdade de Medicina, Universidade de São Paulo, São Paulo, Brazil; ^4^ Laboratory of Toxicant and Drug Analyses - LATF, Federal University of Alfenas - Unifal-MG, Alfenas, MG, Brazil; ^5^ John Jay College of Criminal Justice, City University of New York, New York City, GA, United States; ^6^ Department of Clinical and Toxicological Analyses, School of Pharmaceutical Sciences, University of São Paulo, São Paulo, Brazil

**Keywords:** nicotine metabolite ratio, varenicline, bupropion, smoking cessation, trans-3'hydroxycotinine, cotinine

## Abstract

**Background:** Smoking is the leading cause of preventable death worldwide. It is responsible for several types of cancer, cardiovascular diseases, and diseases of the reproductive system, among others. Therefore, advances in research are increasingly necessary in order to make smoking cessation treatment more effective. Some studies have investigated the association of the nicotine metabolite ratio (NMR) with general characteristics and treatment outcomes. In the present study, the main aim was to evaluate the NMR in smoking patients from an Assistance Program of a tertiary cardiology hospital.

**Methodology:** Serum samples were collected from 185 patients at T0 (while patients were still smoking and before starting pharmacological treatment). Cotinine and hydroxycotinine analytes were measured using liquid-chromatography tandem mass-spectrometry (LC-MS/MS). By looking at the relationship between hydroxycotinine and cotinine, we can obtain the NMR, with which it is possible to classify patients into slow metabolizers (NMR < 0.31), as well as normal or fast metabolizers (NMR ≥ 0.31).

**Results:** From 185 patients, 55 were considered slow metabolizers and 130 as normal/fast. The metabolite averages were associated with the number of cigarettes smoked per day (*p* < 0.001 for cotinine and 0.023 hydroxycotinine). However, we were unable to analyze the association of the NMR with general and clinical characteristics of patients under smoking cessation treatment.

**Conclusion:** We were able to evaluate the NMR, and to observe categories of metabolizers in Brazilian patients under pharmacological treatments. Thus, this study can contribute to the indication of a form of analysis, which might form part of the customization of smoking cessation treatments and, consequently, improve the success rates.

## 1 Introduction

Nicotine is metabolized mainly by cytochrome p450 (CYP) 2A6, and to a lesser extent by enzymes CYP2B6, CYP2D6, and CYP2E1 (Messina et al., 1997; Allenby et al., 2016). Through a CYP2A6-mediated metabolism, cotinine (CO) is the primary metabolite of nicotine, which is still metabolized in 3'-hydroxycotinine (3HC). This pathway represents 70–80% of nicotine metabolism, where cotinine metabolites represent the majority of urinary metabolites (Allenby et al., 2016). Cotinine’s half-life is approximately 13–19 h, which is much longer than the half-life nicotine (1–2 h) or 3HC (approximately 5 h) (Mamoun et al., 2015; Allenby et al., 2016). Because CO has a long half-life, its concentrations in the blood and urine of smokers are relatively stable throughout the day. However, they are still somewhat dependent on time of the last cigarette (Benowitz & Jacob, 2001; Allenby et al., 2016). Thus, 3HC concentrations depend on CO metabolism, which is mediated by CYP2A6 49. The ratio of 3HC to CO is a stable measure of activity of CYP2A6 and this does not depend on the time of the last nicotine intake.

Nicotine Metabolite Ratio (NMR) is a validated biomarker of nicotine metabolism. It is calculated using the 3HC/CO ratio. Studies indicate that higher rates are associated with greater nicotine metabolism (≥0.31) and longer abstinence with non-nicotinic drugs, while lower rates indicate less nicotine metabolism (<0.31), allowing for longer abstinence in smoking cessation treatment with nicotinic repositories (Lerman et al., 2006; Schnoll et al., 2009; Allenby et al., 2016). NMR can be reliably measured in saliva, plasma or serum, has minimal daytime variation, and does not depend on the time that the last cigarette was smoked for those individuals who smoke more than five cigarettes a day (Dempsey et al., 2004; Allenby et al., 2016).

Several studies have shown associations between NMR and response to pharmacological treatment for tobacco dependence and smoking cessation (Lerman et al., 2006; Ho et al., 2009; Schnoll et al., 2009; Siegel et al., 2020a) which nowadays include varenicline (a partial agonist of nAChR), bupropion (a norepinephrine reuptake inhibitor, and dopamine and nicotinic antagonist) and nicotine replacement therapy (Slemmer et al., 2000; Fiore et al., 2008). Siegel et al. analyzed NMR as a biomarker of individual differences in nicotine metabolism, the relationship between the NMR and smoking behavior, the clinical utility of using the NMR to personalize treatments for quitting smoking, and the potential mechanisms underlying this relationship (Siegel et al., 2020a). Lerman et al. observed the association between nicotine metabolism rate and response to pharmacological treatment for smoking cessation, in a trial with nicotine label compared to nicotine nasal spray (Lerman et al., 2006). Schnoll et al. study NMR data in a clinical study of 568 smokers with the treated nicotine patch. The authors identified significantly higher dropout rates at the end of treatment for participants in the first NMR quartile (the slowest metabolizers) in compared to all other quartiles (Schnoll et al., 2009). In a study carried out by Ho et al., with smokers African-Americans randomly assigned to receive nicotine or placebo gum and counseling, similar results were also observed: at the end of treatment, there was a trend towards a successful dropout among slower metabolizers compared to normal or fast metabolizers (Ho et al., 2009).

In view of the serious consequences of smoking due the strong possibility of nicotine dependence, the evaluation of relationship between NMR and treatment outcome may contribute to the knowledge of these variables in the Brazilian population and possibly provide support for the smoking cessation process. Due to the evidence that NMR can be a useful tool in personalizing treatment for smoking cessation, the present article aimed to quantify the NMR in smokers treated with varenicline and/or bupropion from an assistance program of a tertiary cardiology hospital. It also sought to evaluate possible associations between the nicotine metabolic profile and the outcomes of the pharmacological treatment.

## 2 Methods

### 2.1 Study population

For this study, 185 patients of both genders, aged ≥ 18, participating in the Smoker Assistance Program of the Instituto do Coração from Hospital das Clínicas/University of São Paulo (HC/FMUSP). The inclusion of patients took place through the research protocol: “Efficacy of the use of genetic markers in the choice of pharmacological treatment for smoking”, which was approved in the submission process to the Institutional Ethics Committee (CAAE: 60133816.0.0000.0068)—SDC: 4341/16/007. Patients who have suffered liver, kidney and gastrointestinal disorders that compromise the metabolism and administration of the drug; patients who used inhibitors or inducers drugs of cytochrome P450 enzymes in the last 6 weeks; alcoholic patients and illicit drug users; patients with unstable psychiatric illnesses; women at risk of pregnancy and patients with contraindications to treatment with bupropion or varenicline were excluded from the study.

The study design consisted of an initial medical visit and an average of four follow-up medical visits that were spread over 12 weeks, performed by physicians specializing in the smoking cessation process. For patients who did not attend the scheduled medical appointments, follow-up was carried out through telephone calls. During consultations, demographic, socioeconomic, and clinical data were collected.

For patients who smoked less than one pack of CPD, a 12-week treatment with Bupropion 150 mg, once a day was indicated for the first 3 days, and on the fourth day the dose was increased to 150 mg, twice a day. For those patients who smoked one or more packs of CPD or for those resistant or relapsed in previous smoking cessation attempts during treatment with bupropion and/or nicotine replacement therapy (NRT), treatment for 12 or 24 weeks was indicated, with varenicline 0.5 mg, once a day for the first 3 days, on the fourth day the dose was increased to 0.5 mg, twice a day and on the seventh day 1 mg, twice a day. Finally, for those patients who did not achieve complete abstinence after two or 3 months of initiating varenicline therapy or who achieved complete abstinence but experienced moderate or severe withdrawal symptoms with varenicline monotherapy, treatment was instituted. with both drugs, bupropion and varenicline.

### 2.2 Nicotine metabolite ratio

The serum samples of participants were collected at time 0 (T0), i.e., before starting pharmacological treatment, while the patient was still smoking. From the sample collected at T0, it was possible to quantify the NMR, using liquid-chromatography tandem mass-spectrometry (LC-MS/MS) instrumentation, and use those results to classify patients into slow metabolizers (NMR < 0.31 ng/ml) and normal or fast metabolizers (NMR ≥ 0.31 ng/ml) (Allenby et al., 2016). Subsequently, the treatment outcome was evaluated at time 4 (T4) and 12 (T12), 4 and 12 weeks after the beginning of pharmacological treatment, respectively. Patients who had stopped smoking at the evaluated times were considered successful. If patients who started treatment with varenicline in monotherapy were considered resistant at T4, they had bupropion as an adjuvant drug. Patients evaluated at T4 and T12 are the same.

Analyses were performed on a Waters (Milford, United States) Acquity UPLC liquid chromatography system coupled to a Micromass Quattro Premier XE mass spectrometer (Wilmslow, United Kingdom). Chromatographic separation was achieved using an Acquicty UPLC BEH C18 column (2.1 × 100 mm, 1.7 µm), also purchased from Waters (Milford, United States). Sample extraction consisted of a very simple and straightforward protein precipitation using a cold mixture of ACN/MeOH (80:20/v:v). For this, multi-tube vortex equipment, model VX-2500, from VWR (Thorofare, United States) was used, as well as a centrifuge, model 5702, from Eppendorf (Berzdorf, Germany). The Microsoft Excel® 2011 program was used to process the data obtained by exporting raw data.

### 2.3 Statistical analysis

Regarding general characteristics, continuous variables (age in years and number of cigarettes per day) were presented as mean and standard deviation, and categorical variables (gender and self-declared race/color) as frequencies.

The chi-square test was performed for a comparative analysis of general categorical variables with the metabolism profile, and frequency of slow metabolizers according to the pharmacological treatment and its outcomes. Shapiro–Wilk and Kolmogorov–Smirnov tests were performed to verify the normality of continuous variables. General characteristics were in accordance with the metabolization profile, and NMR values and metabolites concentration were in accordance with the pharmacological treatment and its outcomes. The Mann–Whitney hypothesis test was used for NMR values and metabolite concentration, as these variables are not normally distributed and therefore were presented as median and interquartile range between 25 and 75%. Spearman's correlation test was performed to assess the association of metabolite concentrations with the number of cigarettes per day.

All statistical analysis was performed using SPSS software (version 20, IBM Corp, Armonk, New York, United States), considering a significance level of *p* < 0.05.

## 3 Results

### 3.1 General characteristics and nicotine metabolite ratio

From the 185 patients evaluated, 130 (70.3%) were classified as normal or fast metabolizers, and 55 (29.7%) as slow metabolizers according to the NMR. The mean age was 51 ± 11 years, 113 (61,1%) being female and 145 (78,4%) self-declared white. The average number of cigarettes per day was 20 ± 9. Table 1 shows the comparison of age, gender, self-declared race/color and number of cigarettes per day according to NMR categories. There were no significant differences between the general characteristics and normal/fast and slow metabolizers.

**TABLE 1:** General characteristics of the total group of patients, according to NMR.

	Normal and fast metabolizers^a^ (*n* = 130)	Slow metabolizers^b^ (*n* = 55)	*p*-value
Age (years)	52 ± 11	50 ± 12	0.32
Gender, female (%)	58.5	67.3	0.26
Self-declared race/color, white (%)	80.0	74.5	0.41
Number of cigarettes per day	20 ± 9	20 ± 8	0.97

a Normal and fast metabolizers = NMR ≥0.31.

b Slow metabolizers = NMR <0.31.

NMR, nicotinic metabolite ratio.

The Sperman correlation test showed that there is a positive correlation between the number of cigarettes smoked per day and the concentration of CO (r = 0.30; *p* < 0.001) and 3HC (r = 0.17; *p* = 0.02) before starting the treatment (T0).

### 3.2 Nicotine metabolite concentration and treatment outcomes

The concentration of nicotine metabolites—CO and 3HC—measured at T0, was presented as median (25 and 75% percentiles). Such concentrations are available in Table 2 according to the results of treatment for smoking cessation, evaluated at 4 and 12 weeks after the start of pharmacological treatment. The NMR values were also presented as median (25 and 75% percentiles). For this calculation, hydroxycotinine was considered as trans-3-hydroxycotinine, since the amount of other isomers was irrelevant.

**TABLE 2:** Concentration of analytes according to outcome in patients treated with drugs for smoking cessation.

	Resistant T4^a^ (*n* = 105)	Success T4^a^ (*n* = 59)	*p*-value	Resistant T12^a^ (*n* = 61)	Success T12^a^ (*n* = 101)	*p*-value
Cotinine (μg/L)^b^	74.60 (47.75–118.53)	59.20 (43.00–92.10)	0.08	75.00 (53.85–120.79)	65.40 (42.45–107.85)	0.11
Hydroxycotinine (μg/L)^b^	60.80 (5.80–101.77)	37.40 (9.60–66.50)	0.07	56.25 (9.75–103.55)	44.60 (6.95–87.00)	0.42
NMR	0.69 (0.09–1.15)	0.60 (0.14–0.93)	0.33	0.65 (0.23–0.96)	0.64 (0.10–1.09)	0.80
Slow metabolizer (%)^c^	28.6	30.5	0.79	27.9	30.7	0.70

a T4 outcome (*n* = 164), T12 outcome (*n* = 162).

b Concentrations of metabolites from serum samples collected before the start of treatment (T0).

c Slow metabolizer (NMR <0.31).

T4, Visit 4 weeks after pharmacological treatment; T12, Visit 12 weeks after pharmacological treatment; NMR, nicotinic metabolite ratio.

From the 164 patients evaluated at T4, only 59 (36%) achieved therapeutic success with smoking cessation pharmacological treatment. Of the 105 (64%) patients who were not successful, and therefore were considered resistant, 30 (28.6%) were slow metabolizers, according to the NMR classification performed at T0. From the 162 patients evaluated at T12, 101 (62.4%) achieved therapeutic success with smoking cessation pharmacological treatment. Of the 61 (37.6%) patients considered resistant, 17 (27.9%) were slow metabolizers, according to the NMR classification performed at T0. No significant differences were identified between the groups evaluated.

Supplementary Table S1 also shows the median nicotine metabolite concentrations (25 and 75% percentiles) that were collected at T0 according to the outcome of treatment with the drugs varenicline, bupropion, and varenicline plus bupropion. None of the metabolites showed a significant difference between the drugs and associated outcomes.

## 4 Discussion

Tobacco use is still a global public health issue and continues to represent the largest cause of preventable death, accounting for nearly 30% of cancer-related mortality in the United States (Lortet-Tieulent et al., 2016). Nowadays, approved smoking cessation drugs such as bupropion and varenicline have a success rate of about one third of patients (Cahill et al., 2013; Hartmann-Boyce et al., 2014; Kotz et al., 2014), while nicotine replacement therapy presents rates of 20–25% (Stead et al., 2012). In this context, where only about a third of patients are successful with treatment, the use of strategies that allow individualized treatment, such as those that consider genetic and/or individual factors, may be able to increase success rates (King et al., 2012).

NMR has been validated as a tool to select the optimal treatment for smokers to ensure the best possible outcomes (Siegel et al., 2020b), as the 3HC/CO ratio is expected to reflect CYP2A6 activity. Therefore, it is possible to infer that the greater the activity of CYP2A6, the greater the nicotine intake to maintain the desired levels of the substance in the body, which, consequently, would lead to the need to smoke more cigarettes (Allenby et al., 2016).

The relationship between NMR and response to pharmacological treatment of smoking may be mediated by variability in the neurobiological reward of nicotine. Fast nicotine metabolizers have greater availability of nicotinic receptors, which leads to increased brain activity in response to smoking and greater subjective reward. These patients exhibit more signs and symptoms of nicotine dependence and titrate their nicotine dose by smoking more cigarettes per day (Siegel et al., 2020b). Our analysis was unable to identify differences between slow and fast or normal metabolizers in terms of the number of CPD—an average of 20 cigarettes was identified in both groups. However, a positive correlation between the number of CPD and the concentration of CO and 3HC was found. This data corroborates studies that show greater confidence in NMR measurements from plasma samples (Tanner et al., 2015).

In our study, 29.7% of patients were classified as slow metabolizers according to the NMR and no significant differences were found between NMR and general characteristics. Some studies have also failed to demonstrate significant associations between NMR and gender, age or race (St.Helen et al., 2019; Verplaetse et al., 2020; Vogel et al., 2021). On the other hand, there is evidence that higher NMR was associated with female gender, white race, number of cigarettes per day and measures of nicotine dependence (Chen et al., 2018).

Regarding the response to pharmacological treatment, studies have shown that bupropion significantly increased dropout rates among fast nicotine metabolizers, but did not provide any additional benefit to slow metabolizers (Patterson et al., 2008). Although it was not possible to detect significant differences, our study found that only 14.3% of slow metabolizers treated with bupropion achieved success after 12 weeks of treatment. This was the lowest success rate among slow metabolizers found in our analysis. Other studies have also shown a higher success rate among fast metabolizers when treated with varenicline, suggesting that nicotine replacement therapies are the most appropriate treatment for slow metabolizers (Glatard et al., 2017; Lerman et al., 2015).

Shahab et al. failed to demonstrate differences between varenicline and NRT therapy according to NRM in the real-world setting. Some factors must be considered when trying to replicate clinical trial findings in real-world studies, such as sociodemographic differences and ethnic variations among participants included in randomized controlled trials and population studies. In addition, rigorous monitoring of treatment adherence is often difficult to perform outside of controlled environments. Finally, clinical trials will aim to maximize follow-up response to obtain an accurate estimate of treatment effect, whereas follow-up rates in population studies like ours generally tend to be lower, potentially resulting in underestimations of treatment effects in the context of intention-to-treat analysis (Shahab et al., 2019).

It should also be considered there is a substantial variation in the response to pharmacological treatment for smoking cessation. Evidence suggests that individual and environmental factors may contribute to these differences. NMR has proved to be a useful tool that contributes to better pharmacological responses. In a context of personalized medicine, NMR could be applied in clinical practice, in a way that makes it possible to quantify analytes and calculate NMR in the medical consultation, so that the best medication for each patient is selected. Unfortunately, due to the sample size, we were not able to demonstrate significant associations between NMR and general characteristics or the outcome of pharmacological treatment—this being the main limitation of our study. Nevertheless, we believe that the use of NMR shows great potential and can have a synergistic effect on smoking cessation, along with new behavioral approaches to adherence enhancement and pharmacogenetic tools, and should be the focus of future research in this area.

## 5 Conclusion

We were able to evaluate NMR, and to observe categories of metabolizers, in Brazilian patients under pharmacological treatments. Thus, this study can contribute to the indication of a form of analysis, which might form part of the customization of smoking cessation treatments and, consequently, improve success rates.

## Data Availability

The original contributions presented in the study are included in the article/Supplementary Material, further inquiries can be directed to the corresponding author.
