# Supplementary material for: Evaluation of the nicotine metabolite ratio in smoking patients treated with varenicline and bupropion
Source: Front Pharmacol. 2022 Jul 22;13:900112. doi: 10.3389/fphar.2022.900112 (PMC9354088; doi:10.3389/fphar.2022.900112)
Supplement: Supplementary file 1 [file Table1.DOCX]

Supplementary Material

**Table 1: Concentration of analytes according to the outcome of treatment with varenicline, bupropion, and varenicline plus bupropion.**

| **Varenicline** | | | | | | |
| --- | --- | --- | --- | --- | --- | --- |
|  | **Resistant T4**  **(n = 70)** | **Success T4**  **(n = 46)** | **P-value** | **Resistant T12**  **(n = 8)** | **Success T12**  **(n = 56)** | **P-value** |
| **Cotinine (**μ**g/L)** | 71.10 (47.50 - 104.05) | 62.30 (41.23 - 86.93) | 0.17 | 75.35 (58.48 - 158.49) | 65.10 (41.80 - 102.98) | 0.22 |
| **Hydroxycotinine (**μ**g/L)** | 59.70 (11.55 - 98.35) | 37.65 (10.50 - 69.32) | 0.23 | 55.50 (27.85 - 81.43) | 44.77 (11.15 - 78.32) | 0.56 |
| **NMR** | 0.67 (0.20 - 1.20) | 0.61 (0.29 - 0.92) | 0.51 | 0.63 (0.36 - 0.87) | 0.61 (0.29 - 0.93) | 0.97 |
| **Slow metabolizer (%)*** | 28.6 | 28.3 | 0.97 | 12.5 | 26.8 | 0.67 |
| **Bupropion** | | | | | | |
|  | **Resistant T4**  **(n = 31)** | **Success T4**  **(n = 9)** | **P-value** | **Resistant T12**  **(n = 1)** | **Success T12**  **(n = 7)** | **P-value** |
| **Cotinine (**μ**g/L)** | 105.48 (58.00 - 139.64) | 56.10 (41.30 - 135.60) | 0.26 | 39.60 | 56.10 (43.00 - 145.34) | 0.50 |
| **Hydroxycotinine (**μ**g/L)** | 67.23 (2.00 - 119.30) | 40.60 (11.15 - 82.40) | 0.42 | 40.10 | 42.90 (21.10 - 98.66) | 0.75 |
| **NMR** | 0.75 (0.04 - 1.14) | 0.78 (0.17 - 1.17) | 0.78 | 1.01 | 0.78 (0.31 - 1.32) | 0.75 |
| **Slow metabolizer (%)*** | 25.8 | 22.2 | 1.00 | 0.0 | 14.3 | 1.00 |
| **Varenicline and Bupropion** | | | | | | |
|  | **Resistant T4**  **(n = 3)** | **Success T4**  **(n = 4)** | **P-value** | **Resistant T12**  **(n = 27)** | **Success T12**  **(n = 38)** | **P-value** |
| **Cotinine (**μ**g/L)** | 45.90 (41.90 - 58.68) | 64.65 (51.95 - 224.66) | 0.40 | 84.40 (60.92 - 120.30) | 70.30 (41.55 - 108.98) | 0.17 |
| **Hydroxycotinine (**μ**g/L)** | 71.44 (1.60 - 103.67) | 12.80 (0.95 - 34.17) | 0.23 | 59.10 (1.10 - 114.50) | 48.50 (1.10 - 100.10) | 0.66 |
| **NMR** | 1.00 (0.04 - 1.98) | 0.08 (0.01- 0.41) | 0.23 | 0.65 (0.02 - 0.93) | 0.67 (0.02 - 1.29) | 0.56 |
| **Slow metabolizer (%)*** | 33.3 | 75.0 | 0.49 | 33.3 | 39.5 | 0.61 |

T4: Visit 4 weeks after pharmacological treatment; T12: Visit 12 weeks after pharmacological treatment; NMR: Nicotinic metabolite ratio. *Slow metabolizer (NMR <0.31). For varenicline: T4 outcome (n = 116), T12 outcome (n = 64). For bupropion: T4 outcome (n = 40), T12 outcome (n = 8). For varenicline and bupropion: T4 outcome (n = 7), T12 outcome (n = 65)
